# T1 and T2 mapping for identifying malignant lymph nodes in head and neck squamous cell carcinoma

**DOI:** 10.1186/s40644-023-00648-6

**Published:** 2023-12-17

**Authors:** Jiangming Qu, Boju Pan, Tong Su, Yu Chen, Tao Zhang, Xingming Chen, Xiaoli Zhu, Zhentan Xu, Tianjiao Wang, Jinxia Zhu, Zhuhua Zhang, Feng Feng, Zhengyu Jin

**Affiliations:** 1 Department of Radiology, Peking Union Medical College Hospital, Chinese Academy of Medical Sciences, Peking Union Medical Union Medical College, No.1 Shuaifuyuan Wangfujing Dongcheng District, Beijing, 100730 China; 2 Department of Pathology, Peking Union Medical College Hospital, Chinese Academy of Medical Sciences, Peking Union Medical Union Medical College, No.1 Shuaifuyuan Wangfujing Dongcheng District, Beijing, 100730 China; 3 Department of Stomatology, Peking Union Medical College Hospital, Chinese Academy of Medical Sciences, Peking Union Medical Union Medical College, No.1 Shuaifuyuan Wangfujing Dongcheng District, Beijing, 100730 China; 4 Department of Otolaryngology, Peking Union Medical College Hospital, Chinese Academy of Medical Sciences and Peking Union Medical Union Medical College, No.1 Shuaifuyuan Wangfujing Dongcheng District, Beijing, 100730 China; 5 MR Research Collaboration, Siemens Healthineers Ltd, Beijing, China

**Keywords:** T1 mapping, T2 mapping, Diffusion weighted imaging, Head and neck squamous cell carcinoma, Lymph nodes, Metastatic

## Abstract

**Background:**

This study seeks to assess the utility of T1 and T2 mapping in distinguishing metastatic lymph nodes from reactive lymphadenopathy in patients with head and neck squamous cell carcinoma (HNSCC), using diffusion-weighted imaging (DWI) as a comparison.

**Methods:**

Between July 2017 and November 2019, 46 HNSCC patients underwent neck MRI inclusive of T1 and T2 mapping and DWI. Quantitative measurements derived from preoperative T1 and T2 mapping and DWI of metastatic and non-metastatic lymph nodes were compared using independent samples t-test or Mann–Whitney U test. Receiver operating characteristic curves and the DeLong test were employed to determine the most effective diagnostic methodology.

**Results:**

We examined a total of 122 lymph nodes, 45 (36.9%) of which were metastatic proven by pathology. Mean T2 values for metastatic lymph nodes were significantly lower than those for benign lymph nodes (*p* < 0.001). Conversely, metastatic lymph nodes exhibited significantly higher apparent diffusion coefficient (ADC) and standard deviation of T1 values (T1_SD_) (*p* < 0.001). T2 generated a significantly higher area under the curve (AUC) of 0.890 (0.826–0.954) compared to T1_SD_ (0.711 [0.613–0.809]) and ADC (0.660 [0.562–0.758]) (*p* = 0.007 and *p* < 0.001). Combining T2, T1_SD_, ADC, and lymph node size achieved an AUC of 0.929 (0.875–0.983), which did not significantly enhance diagnostic performance over using T2 alone (*p* = 0.089).

**Conclusions:**

The application of T1 and T2 mapping is feasible in differentiating metastatic from non-metastatic lymph nodes in HNSCC and can improve diagnostic efficacy compared to DWI.

**Supplementary Information:**

The online version contains supplementary material available at 10.1186/s40644-023-00648-6.

## Introduction

Cervical lymph node metastasis in head and neck squamous cell carcinoma (HNSCC) is a crucial prognostic factor, significantly reducing disease-free survival and worsening overall prognosis, and it necessitates more aggressive treatment and follow-up [1, 2]. The management of the neck is complicated in HNSCC since it involves different combinations of treatment strategies, and accurate preoperative evaluation of cervical lymph node metastasis still remains challenging [3–5]. Currently, the gold standard for diagnosing cervical lymph node metastasis remains postoperative pathological examination.

MRI has been proven to be a valuable tool for evaluating nodal staging in HNSCC [6]. However, defining lymph node metastasis on MRI without apparent signs of extranodal extension (ENE) or intranodal necrosis is often challenging due to the less reliable size and morphology criteria. Diffusion-weighted imaging (DWI), a non-invasive imaging technique that characterizes tissues based on the restricted diffusion of water molecules, is a simple sequence included in routine head and neck MRI [7]. Although DWI is an essential functional imaging modality widely used to distinguish between benign and malignant lesions, it has been controversial in differentiating lymph node status in HNSCC [8]. Malignant and benign nodes have been reported to exhibit significant overlap in apparent diffusion coefficient (ADC) values, which can be influenced by various imaging conditions [9]. Therefore, it is necessary to identify a more objective and accurate quantitative method for distinguishing between metastatic and non-metastatic lymph nodes.

Quantitative mapping of longitudinal relaxation time (T1) and transverse relaxation time (T2), initially introduced in studies involving the myocardium [10], musculoskeletal system [11], and central nervous system [12], has increasingly extended to the investigation of various tumors. It allows for visualizing quantitative T1 and T2 relaxation times, providing more reproducible data reflecting intrinsic biological tissue characteristics and microstructural differences within lesions. T1 and T2 mapping techniques have been successfully applied in various types of cancer, including the differential diagnosis of benign and malignant lesions [13, 14] and the prediction of pathologic features of cancer [15, 16]. Recent studies have confirmed the potential clinical utility of T1 and T2 mapping techniques in distinguishing between benign and metastatic lymph nodes in mesenteric lymph nodes for rectal cancer [17] and in retropharyngeal lymph nodes for nasopharyngeal cancer [18]. Based on these promising results, we hypothesize that T1 and T2 mapping can be utilized to differentiate between metastatic lymph nodes and reactive lymph node hyperplasia in HNSCC.

Hence, the main objective of this study was to investigate the clinical applicability of T1 and T2 mapping compared to DWI for distinguishing between benign and metastatic cervical lymph nodes, aiming to achieve more accurate nodal staging in HNSCC.

## Materials and methods

### Patients

**Fig. 1:**
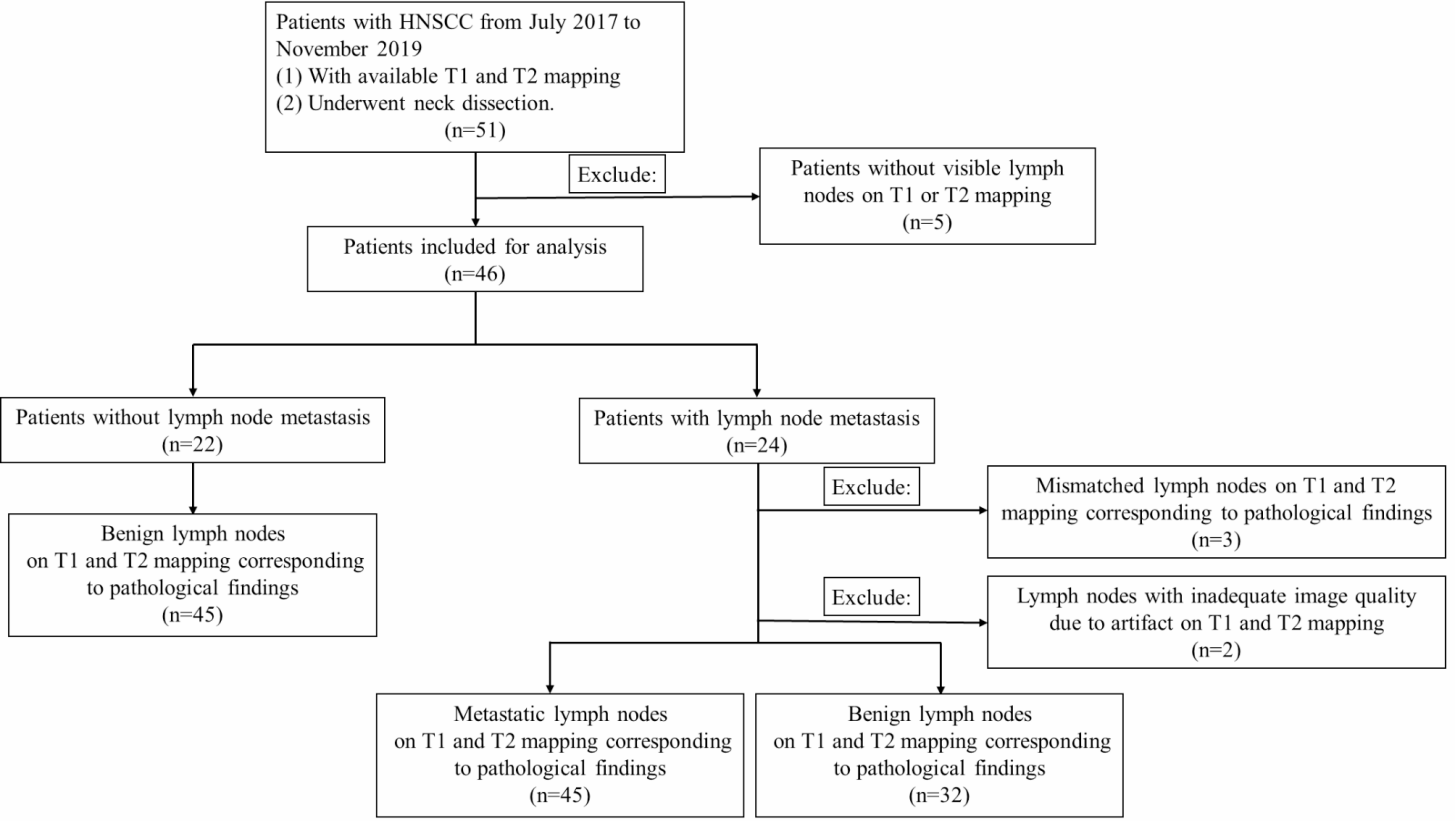
Flow diagram of patient inclusion and exclusion criteria

At Peking Union Medical College Hospital, a total of 51 HNSCC patients were retrospectively enrolled in this study from July 2017 to November 2019. Patients with suspected cervical lymph node metastasis underwent elective neck dissection along with surgical removal of primary cancer based on pre-surgery imaging or clinicopathological high-risk factors. The inclusion criteria for this study involved elective cervical lymph node dissection for HNSCC, as well as the requirement for available T1 and T2 mapping data (Fig. 1). Five patients were excluded due to the inability to visualize small lymph nodes on T1 or T2 mapping. The interval between MRI and surgery was within two weeks.

### Imaging protocols

All the examinations were performed on a clinical 3-Tesla scanner (MAGNETOM Skyra, Siemens Healthcare, Erlangen, Germany) with a 20-channel head-neck coil. Images were acquired sequentially for each sequence, with detailed parameters listed in Table 1. T1 mapping was derived from Magnetization Prepared 2 Rapid Acquisition Gradient Echoes (MP2RAGE) sequence with inversion times of 700 ms and 2500 ms, and flip angles of 4° and 5°. The sagittal T1 mapping was reconstructed into axial images for data anlysis. T2 mapping was obtained using a research multi-echo spin echo (MSE) sequence with GRAPPATINI acceleration technique. The GRAPPATINI approach undersamples the k-space, by exploiting data redundancies over the sampled echoes together with conventional parallel imaging. The parallel imaging facor was 2 and the undersampling factor was 5, which resulting a 10-fold acceleration. The T1 and T2 parametric maps were generated inline after data acquisition. The contrast-enhanced T1-weighted Star-volumetric interpolated breath-hold examination (StarVIBE) sequence was performed after administration of gadolinium contrast agent at a weight-adjusted dose of 0.1 mmol/kg at a flow rate of 2 mL/s.

**Table 1:** MRI sequence parameters

	DWI	T1 mapping	T2 mapping	StarVIBE
Orientation TR (ms) TE (ms)	Axial 3140 59	Sagittal 3500 3	Axial 6000 10–160 (16 TEs, ΔTE = 10 ms)	3D 4.5 1.6
FOV (mm) Slice thickness (mm) Matrix b values (s/mm^2^) No. of slices Acquisition time (min:sec)	160 × 160 3.0 110 × 110 0, 800 36 1:59	260 × 244 1.0 256 × 240 - 176 5:47	260 × 244 3.0 320 × 240 - 36 2:32	260 × 260 1.0 224 × 224 - 160 3:48

TE, echo time; TR, repetition time; FOV, field of view

### Image evaluation

The ROI delineation method used the largest circular area within the largest axial section. Necrotic regions, identified by the absence of enhancement in postcontrast StarVIBE images, were excluded during ROI delineation. The long-axial and short-axial diameter of each lymph node was also measured based on the contrast-enhanced T1-weighted StarVIBE sequence. To minimize partial volume artifacts on DWI and T2 mapping images, we included lymph nodes for quantitative analysis if their short-axis diameter was at least 4 mm. Mean T1, mean T2, the standard deviation of T1 measurement (T1_SD_), the standard deviation of T2 measurement (T2_SD_), and ADC values of all recognized lymph nodes on T1 and T2 mapping were recorded. Two lymph nodes were excluded from the analysis due to aliasing artifacts in the lower neck region from two patients (Figure S1).

### Matching with the histopathologic result

The head and neck surgeons in the operating room labeled all neck dissection specimens according to their respective neck levels. The pathologist, blinded to the MRI results, manually identified the lymph nodes within each neck level in the specimen. Each lymph node was microscopically examined to determine the presence or absence of tumor cells. The total number of lymph nodes and metastatic lymph nodes from each neck level was recorded on the pathology report. The pathologist further retrospectively reviewed and recorded the maximum diameter of metastatic lymph nodes. Additionally, the presence or absence of ENE was also examined in each metastatic lymph node. The localization of metastatic lymph nodes on MRI was first determined by each neck level and then matched by the largest diameter of metastatic lymph nodes measured on the contrast-enhanced T1-weighted StarVIBE sequence and on pathologic slices. Lymph nodes were excluded if multiple lymph nodes met the aforementioned conditions. In total, three lymph nodes (one malignant and two benign) from one neck level identified on T1 and T2 mapping were excluded as these lymph nodes had similar sizes, making it challenging to correlate them with the pathologic result.

### Statistical analysis

Statistical analyses were conducted using Statistical Package for the Social Sciences (SPSS, version 26.0, IBM) and R Statistical Software (version 4.3.1), with a value of two-tailed *p* < 0.05 deemed to indicate a significant difference. The quantitative parameters of benign and metastatic lymph nodes were compared via independent samples t-test or Mann-Whitney U test, based on whether the data distribution was normal as determined by the one-sample Shapiro-Wilk test. Receiver operating characteristic (ROC) curves analysis was conducted using various parameters, and the DeLong test was employed to compare the areas under the curves (AUCs). The cutoff values were determined by the optimal cut-point closest to the point (0,1) on the ROC curve. Logistic regression analysis was deployed to construct a multi-parametric diagnostic model.

## Results

The final study cohort comprised 46 patients, including 24 patients with metastatic lymph nodes and 22 without metastatic lymph nodes. The detailed clinical characteristics are presented in Table 2. The primary tumor site distribution displayed a significant difference (*p* = 0.032). A total of 122 lymph nodes were identifiable on both T1 and T2 mapping, and the mean number of lymph nodes identified on T1 and T2 mapping was 2.7 per patient (range 1–7; SD 1.6). Pathological examination revealed 77 lymph nodes (63.1%) to be chronic lymphadenitis and 45 lymph nodes (36.9%) to be metastatic, with 26 of the latter also harboring ENE. Specifically, two benign lymph nodes presented with epithelioid granuloma and one benign lymph node presented with a rare condition of ectopic thyroid follicles without thyroid malignancy [19]. The 122 lymph nodes were distributed among various levels: Level I (n = 32, 26.2%), Level II (n = 62, 50.8%), Level III (n = 21, 17.2%), Level IV (n = 5, 4.1%), and Level V (n = 2, 1.6%). The mean short-axis diameter of the malignant lymph nodes (12.1 ± 7.4 mm) was significantly larger than that of benign lymph nodes (7.4 ± 1.9 mm) (*p* < 0.001).

**Table 2:** Baseline characteristics and demographics of the patient cohort

Patient characteristics	Metastasis (n = 24)	Non-metastasis (n = 22)	*p* values
Age median, range (yrs)	62 (31–82)	60.5 (42–85)	0.379
Gender			0.592
Male	21 (87.5%)	18 (81.8%)	
Female	3 (12.5%)	4 (18.2%)	
Primary tumor site			**0.032**
Oral cavity	11 (45.8%)	14 (63.6%)	0.226
Oropharynx	0 (0%)	1 (4.5%)	0.097
Larynx	2 (8.3%)	5 (22.7%)	0.113
Hypopharynx	11 (45.8%)	2 (9.1%)	**0.006**
pT			0.511
T1	0 (0%)	1 (4.5%)	
T2	8 (33.3%)	9 (40.9%)	
T3	8 (33.3%)	4 (18.2%)	
T4	8 (33.3%)	8 (36.4%)	

**Fig. 2:**
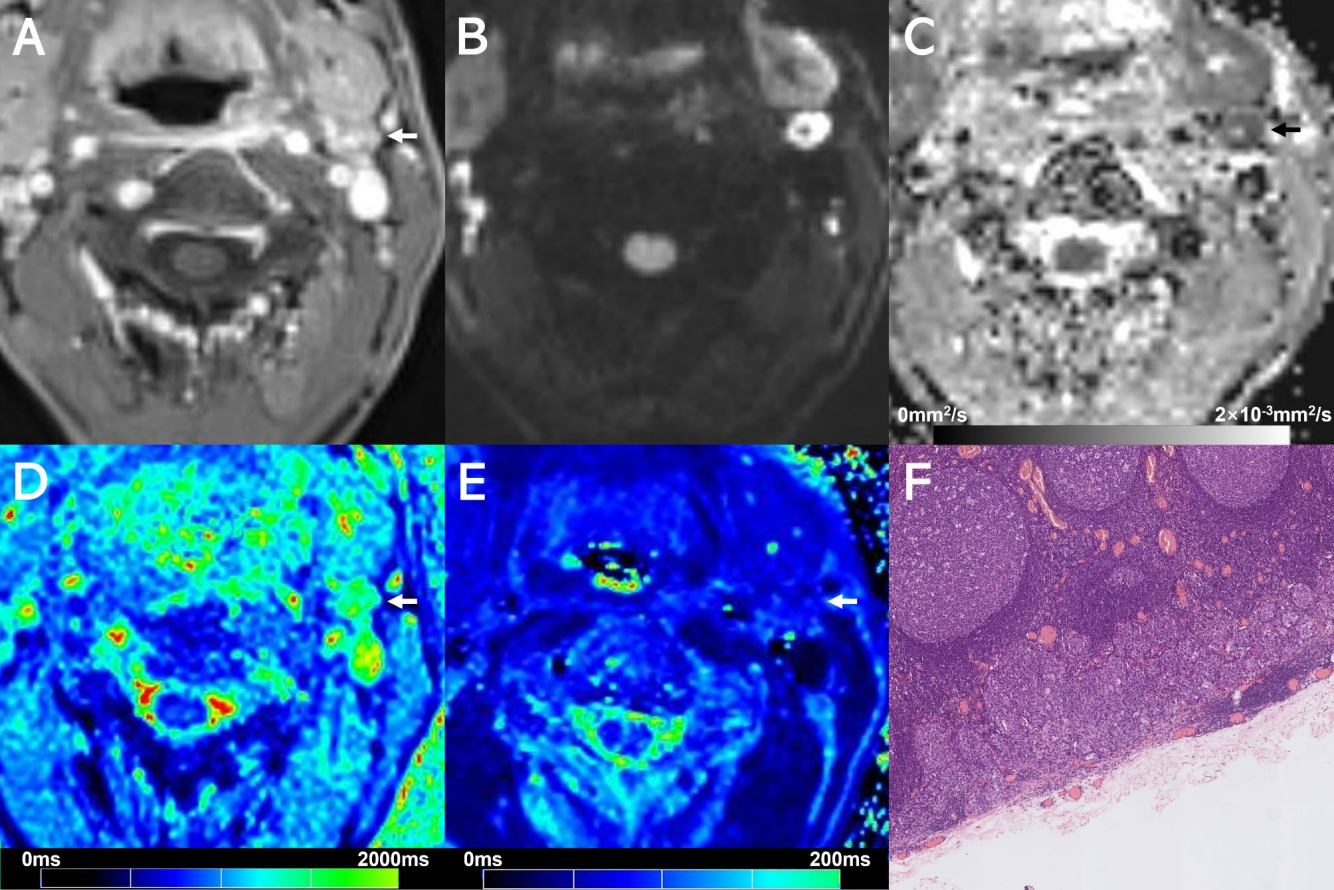
Images show a 7.5-mm size metastatic lymph node (arrows) at left level II from a 49-year-old male with hypopharyngeal squamous cell carcinoma. (**A**) Contrast-enhanced T1-weighted image shows the lymph node. (**B**) DWI with a b-value of 800 mm^2^/s shows a hyperintense area. (**C**) The ADC map on the targeted lymph node shows a hypointense signal, an ADC value of 0.831 × 10^− 3^ mm^2^/s. (**D**) T1 mapping and (**E**) T2 mapping show a T1 value of 1708.2 ± 216.6 ms and a T2 value of 76.6 ± 11.0 ms. (**F**) Section through the same lymph node showing metastatic deposit

**Fig. 3:**
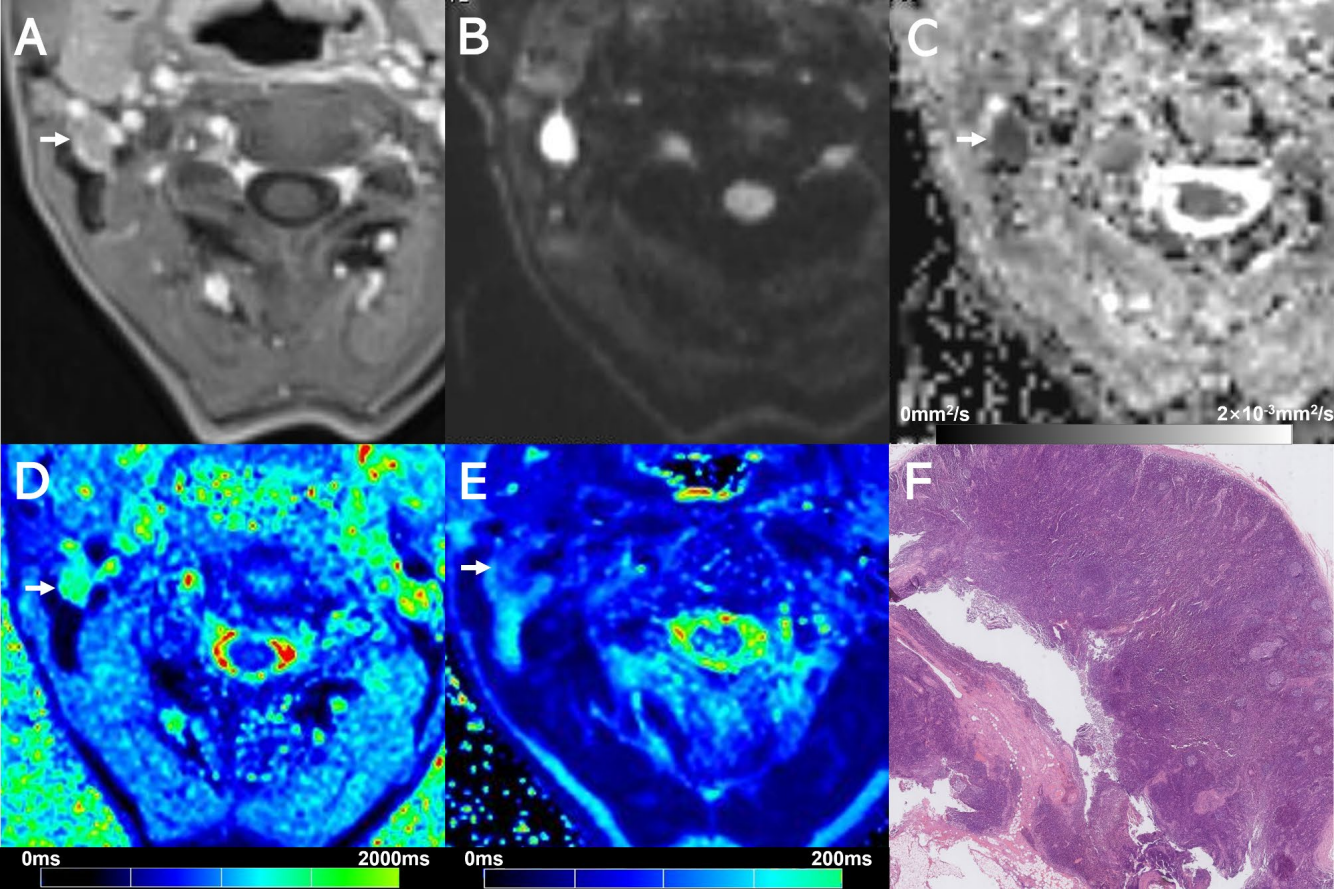
Images show a 7.9-mm size benign lymph node (arrows) at right level II from a 49-year-old male with hypopharyngeal cancer (the same patients in Fig. 2). (**A**) Contrast-enhanced T1-weighted image shows the lymph node (arrow). (**B**) DWI with a b-value of 800 mm^2^/s shows a hyperintense area. (**C**) The ADC map on the targeted lymph node shows a hypointense signal, an ADC value of (0.698 × 10^− 3^mm^2^/s). (**D**) T1 mapping and (**E**) T2 mapping show a T1 value of 1746.1 ± 115.1 ms and a T2 value of 92.0 ± 9.7 ms. (**F**) Section through the same lymph node showing absence of metastasis

**Fig. 4:**
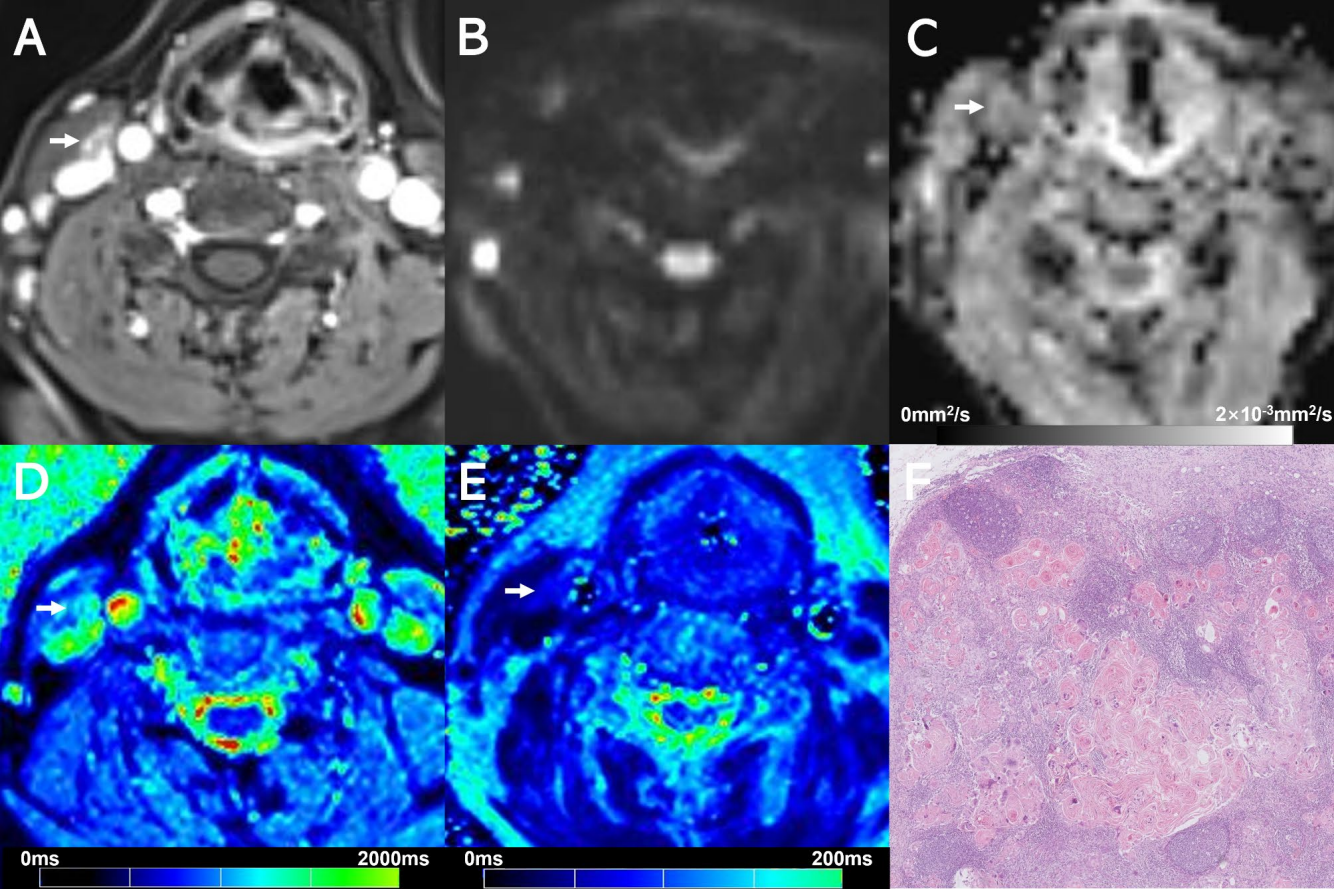
Images show a 5.0-mm size metastatic lymph node (arrows) at right level III from a 63-year-old female with oral cavity squamous cell carcinoma. (**A**) Contrast-enhanced T1-weighted image shows the lymph node (arrow). (**B**) DWI with a b-value of 800 mm^2^/s shows a hyperintense area. (**C**) The ADC map on the targeted lymph node shows a hypointense signal, an ADC value of (1.003 × 10^− 3^mm^2^/s). (**D**) T1 mapping and (**E**) T2 mapping show a T1 value of 1434.3 ± 148.8 ms and a T2 value of 64.4 ± 4.1 ms. (**F**) Section through the same lymph node showing metastatic deposit

**Fig. 5:**
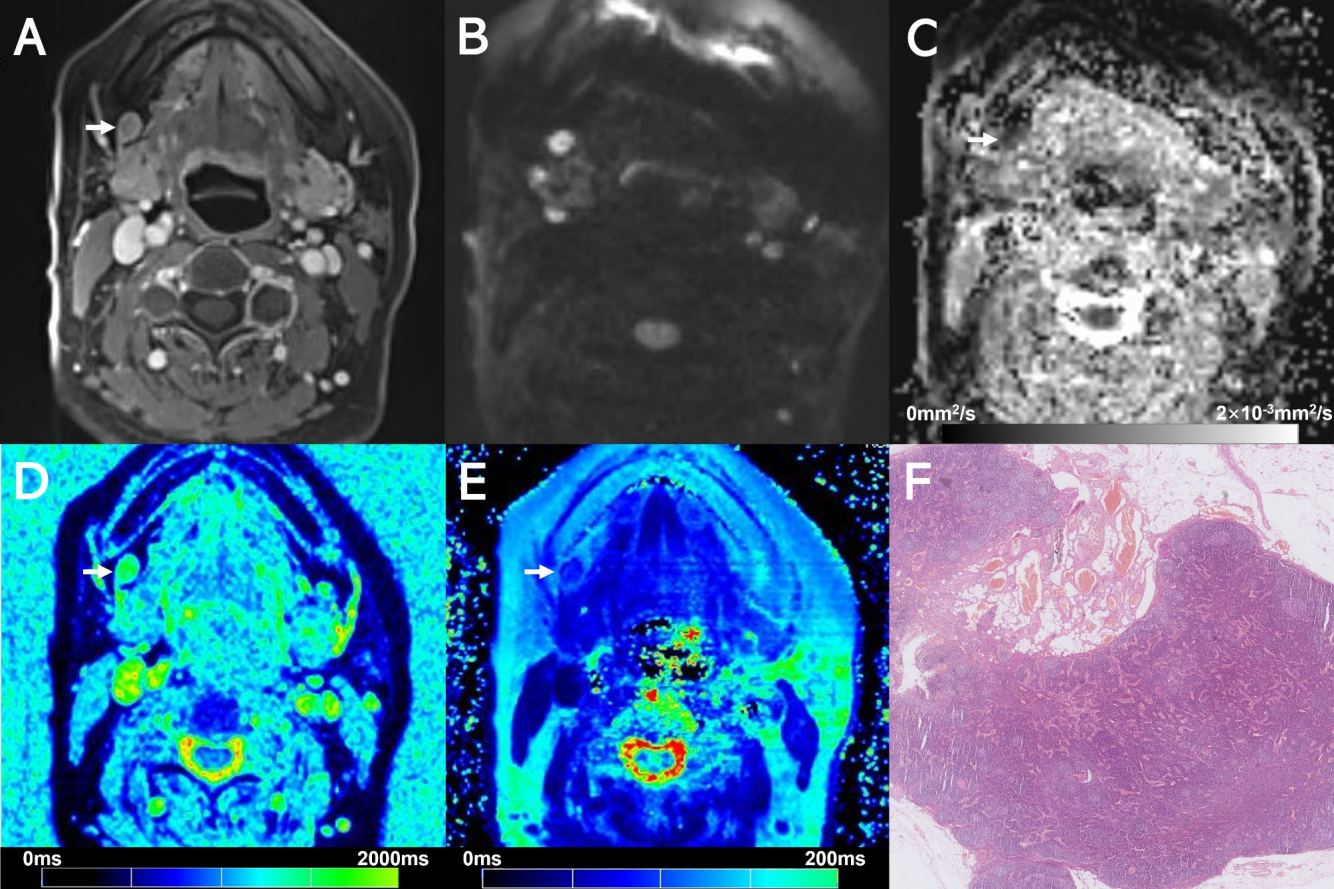
Images show a 7.7-mm size benign lymph node (arrows) at right level I from a 65-year-old female with oral cavity squamous cell carcinoma. (**A**) Contrast-enhanced T1-weighted image shows the lymph node (arrow). (**B**) DWI with a b-value of 800 mm^2^/s shows a hyperintense area. (**C**) The ADC map on the targeted lymph node shows a hypointense signal, an ADC value of (0.644 × 10^− 3^mm^2^/s). (**D**) T1 mapping and (**E**) T2 mapping show a T1 value of 1483.6 ± 106.5 ms and a T2 value of 90.1 ± 5.2 ms. (**F**) Section through the same lymph node showing absence of metastasis

Examples of T1 mapping, T2 mapping, and DWI for benign or malignant cervical lymph nodes are shown in Figs. 2, 3, 4 and 5. T2 and T1_SD_ values were significantly higher in benign lymph nodes compared to malignant lymph nodes, whereas ADC values were significantly lower in benign lymph nodes than in malignant lymph nodes (all *p* < 0.05), as displayed in Table 3. No significant difference was observed in T1 and T2_SD_ values between these two groups (*p* > 0.05). Moreover, metastatic lymph nodes with nodal necrosis had significantly higher T2 than the solid part of metastatic lymph nodes without nodal necrosis (Table 4). None of the parameters showed a significant difference with or without ENE (*p* > 0.05) (Table S1).

**Table 3:** Comparison of ADC, T1, and T2 for non-metastatic and metastatic lymph nodes

Parameters	Benign	Malignant	*p* values
T1 (ms) T1_SD_ (ms) T2 (ms) T2_SD_ (ms) ADC (×10^− 3^ mm^2^/s)	1536.83 ± 145.50 164.71 ± 63.61 95.24 ± 9.10 9.17 ± 3.47 0.87 ± 0.23	1523.53 ± 231.47 224.56 ± 97.73 78.86 ± 10.23 8.81 ± 2.90 0.93 ± 0.14	0.732 **< 0.001** **< 0.001** 0.667 **0.004**

**Table 4:** Comparison of ADC, T1, and T2 for metastatic lymph nodes with or without nodal necrosis

Parameters	Without nodal necrosis	With nodal necrosis	*p* values
T1 (ms) T1_SD_ (ms) T2 (ms) T2_SD_ (ms) ADC (×10^− 3^ mm^2^/s)	1502.53 ± 252.83 213.72 ± 81.18 75.94 ± 8.64 8.29 ± 2.89 0.94 ± 0.15	1565.53 ± 182.06 246.24 ± 124.95 84.71 ± 10.94 9.85 ± 2.73 0.90 ± 0.12	0.563 0.298 **0.005** 0.089 0.443

**Fig. 6:**
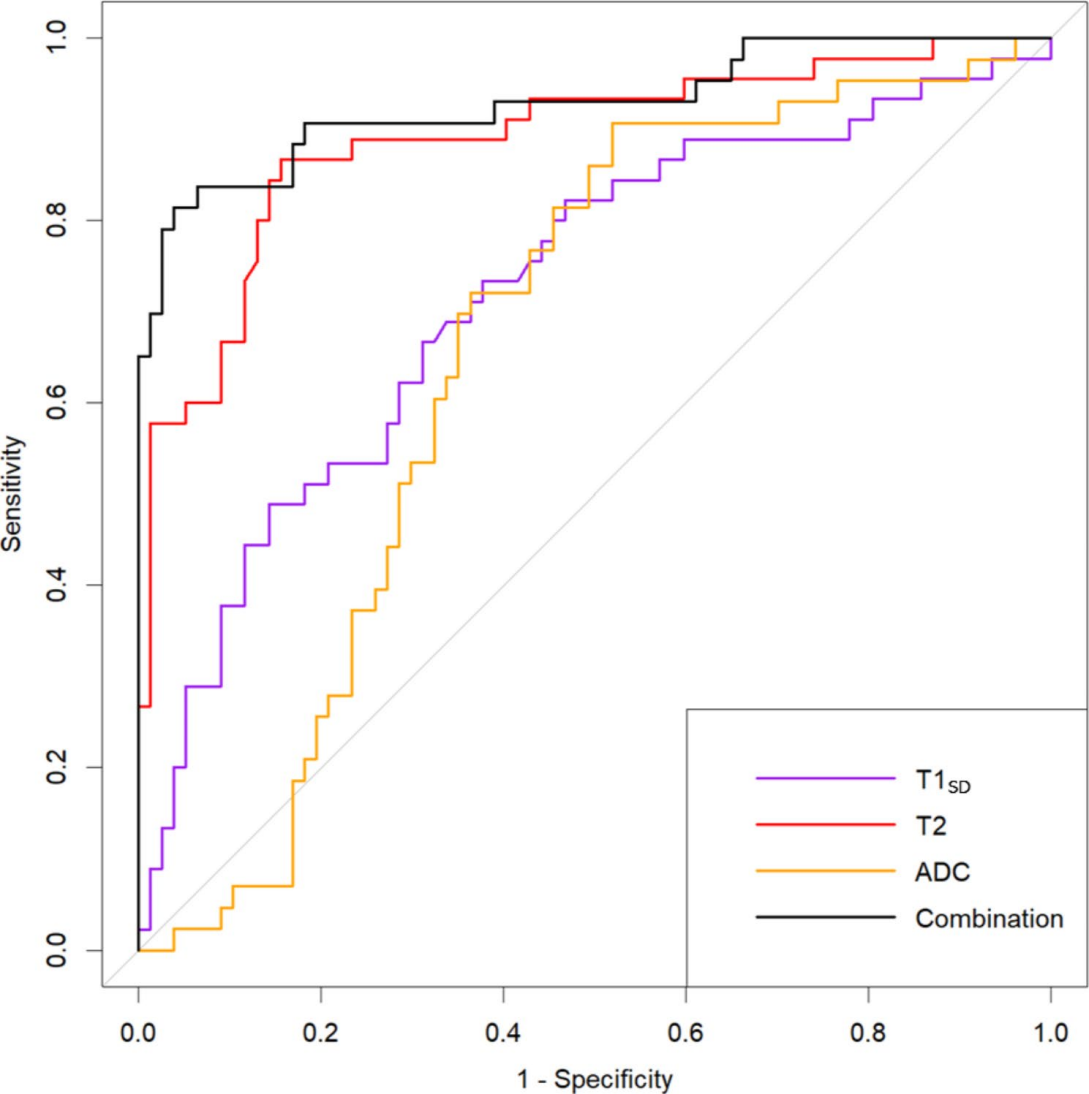
Receiver operating characteristic (ROC) curves for discriminating metastatic from non-metastatic lymph nodes for T1_SD_, T2, ADC, and a combined model. The area under the ROC curve (AUC) of the T2 value was significantly higher than for the ADC value (AUC T2 value = 0.890, AUC ADC value = 0.660, *p* < 0.001)

For all the analyzed lymph nodes, the AUC derived from the ROC curves were 0.890 (95% CI: 0.826–0.954) for T2 value, 0.711 (95% CI: 0.613–0.809) for T1_SD_, and 0.660 (95% CI: 0.562–0.758) for ADC (Fig. 6). T2 demonstrated significantly better diagnostic performance in discriminating between metastatic and non-metastatic lymph nodes compared to ADC (*p* < 0.001) and T1_SD_ (*p* = 0.007). The optimal cutoff value for T2 was determined to be 88 ms, resulting in a sensitivity of 84.4% and specificity of 86.7% for nodal metastasis detection. When combining multiple parameters (T2, T1_SD_, ADC, and short-axis diameter), the AUC increased to 0.929 (95% CI: 0.875–0.983), slightly higher than using the T2 value alone, although the difference was not significant (*p* = 0.089).

We further examined the diagnostic performance of T1 and T2 mapping in differentiating benign or malignant subcentimeter lymph nodes and non-necrotic lymph nodes within our cohorts. For the 88 subcentimeter lymph nodes, the AUC derived from the ROC curves was 0.918 (95% CI: 0.848–0.987) for T2 value, 0.655 (95% CI: 0.505–0.806) for T1_SD_, and 0.711 (95% CI: 0.604–0.817) for ADC, with the AUC of T2 value significantly higher than that of ADC value and T1_SD_ (both *p* = 0.002). For the 107 lymph nodes without nodal necrosis, the AUC derived from the ROC curves was 0.948 (95% CI: 0.907–0.989) for T2 value, 0.694 (95% CI: 0.574–0.815) for T1_SD_, and 0.666 (95% CI: 0.560–0.772) for ADC, with the AUC of T2 value significantly higher than that of ADC value and T1_SD_ (both *p* = 0.002).

## Discussion

In the current study, we delved into the clinical utility of quantitative T1 and T2 mapping to distinguish between benign and metastatic lymph nodes in HNSCC. We observed significant differences in T1_SD_, T2 value, and ADC between metastatic and non-metastatic lymph nodes. T2 value demonstrated superior diagnostic efficacy compared to T1_SD_ and ADC. The optimal T2 threshold was determined to be 88 ms, achieving a sensitivity and specificity of 84.4% and 86.7%, respectively. Adding T1_SD_, ADC, and short-axis diameter to the T2 value did not significantly improve the diagnostic performance beyond the T2 value alone. Similar results were also observed in the subgroup analysis of subcentimeter cervical lymph nodes.

The role of DWI in distinguishing between benign and malignant cervical lymph nodes in HNSCC has been a subject of long-standing controversy. Our study discovered that mean ADC was significantly higher for malignant than benign nodes, albeit with a prominent overlap in distribution. Despite numerous studies presenting a considerably lower ADC value for metastatic lymph nodes compared to benign ones [20–24], several studies have yielded a result similar to our research, substantiating a significantly elevated ADC value for metastatic lymph nodes in HNSCC [25, 26]. A study by Lim et al. found no substantial ADC variation between benign and malignant cervical small lymph nodes [8]. In line with this, Heijnan et al. determined that there was no significant difference in ADC between benign and malignant lymph nodes in rectal cancer [27]. Notably, the ADC values of benign lymph nodes from our study appear comparatively lower than in previous studies. ADC mainly represents water compartmentalization and diffusion and also indicates cellularity. Previous pathology analyses have suggested that ADC is a superior cell density indicator over T2 [28]. Reactive lymph node hyperplasia, characterized by uniform lymphoid infiltration, organized germinal centers, and fibrous stroma, could potentially elevate microstructural barriers [29]. Our results suggest that the use of DWI should be applied with caution and deserves additional verification.

Our study revealed that metastatic lymph nodes exhibit significantly shorter T2 than benign ones, similar to the findings on retropharyngeal lymph nodes and mesenteric lymph nodes [17]. Notably, metastatic lymph nodes without necrosis have even significantly shorter T2 than the non-necrotic part of metastatic lymph nodes with necrosis. The pathophysiology underpinning the diminished T2 value is thought to reflect the reduction in tumor water content and a lower T2 is also associated with an increase in tumor cellularity and necrosis [30–32]. In its initial applications, T2 value was utilized as a biomarker for tumor edema in brain cancer [33]. Subsequent studies in the field of body cancer have affirmed the effectiveness of T2 mapping in differentiating benign from malignant lesions across various tissues, including the prostate, breast, and parotid gland, among others. T2 mapping has also shown potential in predicting histopathological type, grade, and other attributes, in which lower T2 values often correlate with more aggressive histopathological features in renal, cervical, and rectal cancers [34–36]. Experimental tumor models have substantiated that T2 value is a more sensitive measure of tumor water content than ADC [37]. Though the pathophysiological rationale for decreased T2 yet heightened ADC in malignant lymph nodes and the even lowered T2 in malignant lymph nodes without necrosis warrants further investigation, the results of our study showed that T2 significantly surpassed ADC in diagnostic performance, suggesting its feasibility in discerning between benign and malignant lymph nodes. Therefore, T2 mapping may serve as a non-invasive technique for nodal staging in HNSCC.

With respect to T1 mapping, our study identified no significant difference in mean T1 between benign and malignant lymph nodes, yet we registered a remarkably higher T1_SD_ for metastatic lymph nodes. This outcome appears to contrast with findings from the study on retropharyngeal lymph nodes, which reported significantly elevated values for both average T1 and T1_SD_ in benign lymph nodes [18]. At the pathological level, the T1 of experimental tumors has positively correlated with increased tumor water content and cellular proliferation and is negatively correlated with tumor necrosis [31, 32, 38]. The equivalent T1 between metastatic and non-metastatic lymph nodes could possibly result from decreased water content that lowering T1 counteracts with increased cellular proliferation, increasing T1. Within other clinical investigations, a higher T1 has been associated with higher-risk histopathologic features and dismal prognosis [15, 16, 39, 40], with one report noting elevated T1 values for malignant lesions than benign lesions [41]. Additionally, radiological features derived from T1 mapping, as explored through radiomics techniques, have been recently found valuable in predicting Gleason scores in prostate cancer [42] and distinguishing between benign and malignant lesions in the nasopharynx [43]. The significantly higher T1_SD_ for metastatic lymph nodes suggests a more complex heterogeneity inside the metastatic lymph nodes and calls for further exploration of radiomic features on T1 mapping, as mapping methodologies can offer precision on a voxel-by-voxel basis. Despite T1 and T2 mapping, along with DWI, appearing to denote different facets of intrinsic tumor microstructure, the composite use of T1_SD_ or T2 values with ADC did not significantly enhance the diagnostic performance.

A noteworthy aspect of our study design involves the inclusion of both benign and malignant lymph nodes from patients diagnosed with HNSCC. Several studies have shown potential selection bias regarding the lymph nodes subjected to analysis. Since nasopharyngeal cancer is primarily treated non-surgically, the malignancy of enlarged lymph nodes is inferred from traditional radiological findings. The inclusion criteria of Wang et al.‘s study specifically selected enlarged lymph nodes with necrosis or extracapsular nodal spread for analysis [18]. However, the T1 and T2 values of those lymph nodes with ambiguous radiologically malignant features were not investigated. In certain studies, benign lymph nodes selected for analysis were obtained from healthy, non-cancerous patients [18, 23]. Consequently, reactive hyperplastic lymph nodes from patients with cancer were not evaluated. This omission raises the question of whether the quantitative parameters of benign lymph nodes from healthy individuals can accurately represent reactive hyperplastic lymph nodes from cancer patients since regional lymph nodes have been reported to take an active role in anti-cancer immunity [44, 45].

Several limitations of the study must be acknowledged. Firstly, the study population was retrospectively identified from a single center, and larger independent cohorts are necessary to validate these results using the T2 value alone and the combination method. In the future, accelerated T1 and T2 mapping with small slice thickness will enable the visualization and study of lymph nodes smaller than 4 mm in more patients with HNSCC. Secondly, there may be potential errors and bias in correlating and matching the lymph nodes between pathological specimens and radiologic images, even though three lymph nodes with ambiguous matches were excluded from the present study. Lastly, we did not explore the relationship between radiologic parameters and other measurable immunohistochemical indices, which could enhance our understanding of the underlying pathophysiology behind the differences in T1_SD_ and T2 value between lymph nodes with or without metastasis, as well as the differences in T2 values between metastatic lymph nodes with or without necrosis.

## Conclusion

In conclusion, our findings indicate that malignant cervical lymph nodes exhibit significantly lower T2 values and higher T1_SD_ and ADC values compared to benign lymph nodes in HNSCC. T2 mapping has the potential to serve as an in vivo biomarker for distinguishing between metastatic and non-metastatic lymph nodes. This finding has important implications for achieving preoperative high-accuracy nodal staging in HNSCC.

### Electronic supplementary material

Below is the link to the electronic supplementary material.

Supplementary Material 1

## Data Availability

The data used and analyzed during the current study are available from the corresponding author upon reasonable request.

## Abbreviations

AUC: Area under the curve
ADC: Apparent diffusion coefficient
DWI: Diffusion-weighted imaging
ENE: Extranodal extension
FOV: Field of view
HNSCC: Head and neck squamous cell carcinoma
MRI: Magnetic resonance imaging
ROC: Receiver operating characteristic
StarVIBE: Stack-of-stars volumetric interpolated breath-hold examination
TE: Echo time
TR: Repetition time
