# Supplementary material for: T1 and T2 mapping for identifying malignant lymph nodes in head and neck squamous cell carcinoma
Source: Cancer Imaging. 2023 Dec 17;23:125. doi: 10.1186/s40644-023-00648-6 (PMC10726506; doi:10.1186/s40644-023-00648-6)
Supplement: Supplementary file 1 — Supplementary Material 1 [file 40644_2023_648_MOESM1_ESM.docx]

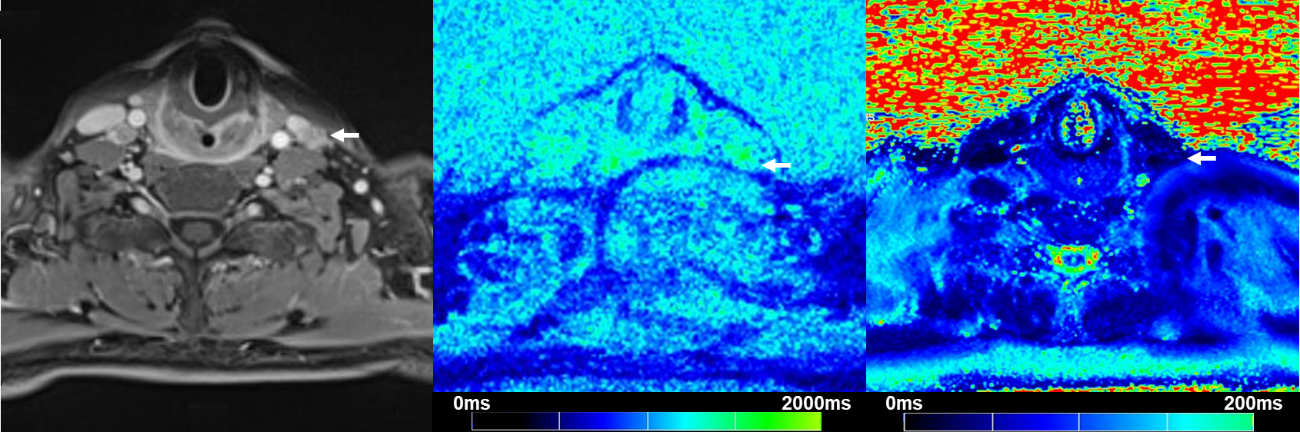


**Fig. S1** Example of excluded lymph nodes with inadequate image quality due to aliasing artifact. The artifact of the shoulder is superimposed on the left level VI lymph nodes (arrow). (A) Contrast-enhanced T1-weighted image. (B) T1 mapping image. (C) T2 mapping image.

**Table S1** Comparison of ADC, T1, and T2 for metastatic lymph nodes with or without ENE

| Parameters | ENE- | ENE+ | *p-*values |
| --- | --- | --- | --- |
| T1 (ms)  T1_SD_ (ms)  T2 (ms)  T2_SD_ (ms)  ADC (×10^-3^ mm^2^/s) | 1498.58±206.30  210.72±116.97  79.48±11.24  8.67±3.19  0.92±0.17 | 1541.77±250.7  234.68±81.90  78.41±9.64  8.91±2.74  0.93±0.11 | 0.535  0.423  0.735  0.787  0.709 |
